# Characterization of a thermophilic and glucose-tolerant GH1 β-glucosidase from hot springs and its prospective application in corn stover degradation

**DOI:** 10.3389/fmicb.2023.1286682

**Published:** 2023-12-21

**Authors:** Yu-Ying Huang, Zhi-Hua Lv, Hong-Zhao Zheng, Qian Zhu, Meng-Ting Liu, Peng Sang, Fei Wang, Dan Zhu, Wen-Dong Xian, Yi-Rui Yin

**Affiliations:** ^1^College of Agriculture and Biological Science, Dali University, Dali, China; ^2^Key Laboratory of Bioinformatics and Computational Biology, Department of Education of Yunnan Province, Dali University, Dali, China; ^3^Marine Microorganism Ecological and Application Lab, Marine Science and Technology College, Zhejiang Ocean University, Zhoushan, China; ^4^Cangshan Forest Ecosystem Observation and Research Station of Yunnan Province, Dali University, Dali, China

**Keywords:** hot spring, β-glucosidase, thermophilic, thermostability, glucose tolerance

## Abstract

**Introduction:**

β-Glucosidase serves as the pivotal rate-limiting enzyme in the cellulose degradation process, facilitating the hydrolysis of cellobiose and cellooligosaccharides into glucose. However, the widespread application of numerous β-glucosidases is hindered by their limited thermostability and low glucose tolerance, particularly in elevated-temperature and high-glucose environments.

**Methods:**

This study presents an analysis of a β-glucosidase gene belonging to the GH1 family, denoted *lqbg8*, which was isolated from the metagenomic repository of Hehua hot spring located in Tengchong, China. Subsequently, the gene was cloned and heterologously expressed in *Escherichia coli* BL21(DE3). Post expression, the recombinant β-glucosidase (LQBG8) underwent purification through a Ni affinity chromatography column, thereby enabling the in-depth exploration of its enzymatic properties.

**Results:**

LQBG8 had an optimal temperature of 70°C and an optimum pH of 5.6. LQBG8 retained 100 and 70% of its maximum activity after 2-h incubation periods at 65°C and 70°C, respectively. Moreover, even following exposure to pH ranges of 3.0–10.0 for 24 h, LQBG8 retained approximately 80% of its initial activity. Notably, the enzymatic prowess of LQBG8 remained substantial at glucose concentrations of up to 3 M, with a retention of over 60% relative activity. The kinetic parameters of LQBG8 were characterized using cellobiose as substrate, with *K*_m_ and *V*_max_ values of 28 ± 1.9 mg/mL and 55 ± 3.2 μmol/min/mg, respectively. Furthermore, the introduction of LQBG8 (at a concentration of 0.03 mg/mL) into a conventional cellulase reaction system led to an impressive 43.7% augmentation in glucose yield from corn stover over a 24-h period. Molecular dynamics simulations offered valuable insights into LQBG8’s thermophilic nature, attributing its robust stability to reduced fluctuations, conformational changes, and heightened structural rigidity in comparison to mesophilic β-glucosidases.

**Discussion:**

In summation, its thermophilic, thermostable, and glucose-tolerant attributes, render LQBG8 ripe for potential applications across diverse domains encompassing food, feed, and the production of lignocellulosic ethanol.

## Introduction

Lignocellulose stands as nature’s most abundant source of renewable energy material. Its extensive biomass and inherent renewability position it as a viable bioenergy alternative to fossil fuels (Ariaeenejad et al., 2020). This resource holds vast potential across a spectrum of domains including energy, chemistry, materials, and related industries (Isikgor and Remzi Becer, 2015). Central to lignocellulose is cellulose, the principal constituent, whose complete hydrolysis necessitates the synergistic interplay of multiple glycoside hydrolases (Rizk et al., 2012). These enzymes encompass endoglucanases (EGL, E.C. 3.2.1.4), exoglucanases (CBH, E.C. 3.2.1.91), and β-glucosidases (BGL, E.C. 3.2.1.21). Among these, β-glucosidase, a pivotal catalyst in the cellulose degradation pathway (Lu et al., 2013), mitigates the inhibitory effects of cello-oligosaccharides and cellobiose on endoglucanases and exoglucanases. Nonetheless, the accumulation of glucose, a product arising from cellulose hydrolysis, hampers the efficacy of β-glucosidase-mediated degradation. As a result, the quest for high-glucose-tolerant β-glucosidases has gained prominence (de Giuseppe et al., 2014). Elevated temperature conditions offer manifold advantages, including augmented enzyme-substrate interaction rates, reduced contamination risks, heightened matrix solubility, and accelerated diffusion rates (Schröder et al., 2014; Ratuchne and Knob, 2021). Consequently, numerous industrial processes are conducted under high-temperature conditions. Pang et al. (2017) underscored the significance of attributes such as thermostability, catalytic efficiency, and glucose tolerance for industrial β-glucosidases. In this context, thermophilic and thermostable β-glucosidases have emerged as pivotal candidates for industrial applications.

β-Glucosidases are distributed across plants, animals, fungi, bacteria, and archaea. Notably, β-glucosidases from thermophilic microorganisms often exhibit heightened thermostability and extreme pH resilience (Ahmed et al., 2018; Martins et al., 2019). Particularly, thermophilic bacteria and archaea sourced from hot springs manifest these attributes (Zayulina et al., 2021). Recent investigations have revealed that over 99% of prokaryotic microorganisms remain elusive through existing pure culture isolation techniques (Tiwari et al., 2021). Metagenomic sequencing technology has emerged as a potent avenue to directly access functional gene sequences from environmental DNA (Kaushal et al., 2021; Robinson et al., 2021; Sousa et al., 2022).

Hot springs harbor a wealth of thermophilic microorganisms and enzymes (Hou et al., 2013), including DNA polymerase, β-galactosidase, esterase, and amylase, which have been gleaned from hot spring metagenomes (Mirete et al., 2016). Prior studies, such as those by Schröder et al. (2014) and Kaushal et al. (2021), have reported the presence of β-glucosidases Bgl1 and BglM derived from hot spring metagenomes; however, such inquiries remain relatively sparse. This study presents a novel perspective, wherein a GH1 family β-glucosidase gene, denoted as LQBG8, was successfully extracted from the Tengchong Hehua hot spring metagenome. Following heterologous expression within *Escherichia coli* BL21(DE3) and purification through a Ni-NTA column, the enzymatic characteristics of LQBG8 were meticulously investigated. The outcomes highlight LQBG8 as a thermophilic β-glucosidase demonstrating resilience to heat, glucose, and a wide pH range. These distinctive attributes underscore its compelling potential across industrial cellulose degradation and production realms.

## Materials and methods

### Sample, medium, strains and plasmid

The sample was collected from the Hehua hot spring (24.90904°N, 98.387627°E), Tengchong City, Yunnan Province, China. The surface temperature of geothermal water was around 65.2°C with pH 8.2. The soil sample was quickly frozen on dry ice for laboratory DNA isolation and metagenome sequencing. Luria-Bertani (LB) medium: 5 g/L yeast extract, 10 g/L trypsin, 10 g/L NaCl, 50 mg/L kanamycin, pH 7.4, solid medium with 2% agar. The pET28a(+) plasmid was used for gene cloning and expression. *E. coli* DH5α and *E. coli* BL21(DE3) were used as host cells for cloning and expression.

### DNA extraction and gene amplification

Total DNA was extracted using the Power Soil Kit (MOBIO DNeasy PowerSoil Kit, United States) according to the manufacturer’s instructions. Metagenomic sequencing was performed using a HiSeq 2500 instrument at GENWIZ, Suzhou. The IMG server^1^ was used to investigate all resulting sequences. To further detect the potential functions of individual genes and ORFs, KEGG, COG, and Pfam were all used. Based on this functional prediction, a β-glucosidase gene sequence—*lgbg8*—was obtained from the metagenomic database.

The above metagenomic DNA was used as a template, and primers (*LQBG8*-F: CAAATGGGTCGCGGATCCGAAATGGCGTT TCTCG CGTTTCCCG and *LQBG8*-R: GTGCTCGAGTGC GGCCGCAAGT CAGACCTC GACCCCGTTCCTG) were used to perform PCR amplification. Underlined sequences represents the homologous recombinant fragment matching the pET28a(+) vector (Novagen, United States). High fidelity DNA polymerase (TransStarFastPfu Fly DNA Polymerase Kit, TransGen Biotech, China) was used for gene amplification. PCR amplification program included pre-denaturation at 94°C for 4 min, followed by denaturation at 94°C for 30 s, annealing at 55°C for 35 s, extension at 72°C for 90 s, 32 cycles and then final extension at 72°C for 5 min.

### Construction and transformation of recombinant plasmid pET28a-lqbg8

The purified PCR product was inserted into the pET28a(+), which had been previously digested by *EcoR I* and *Hind III*, using the pEASY-Uni Seamless Cloning and Assembly Kit (TransGen Biotech, China) to construct the recombinant plasmid *pET28a-lqbg8*. DNA linking products were introduced into *E. coli* DH5α receptor cells by Ca^2+^ chemical transformation method. The positive clones were screened by colony PCR and sequencing, to obtained the recombinant plasmid *pET28a-lqbg8*. The plasmid *pET28a-lqbg8* was transferred to *E. coli* BL21(DE3) for gene expression.

### Sequence analysis

DNA and protein sequences of LQBG8 were compared database sequences by BLASTx and BLASTp programs,^2^ respectively. Signal peptides were predicted using SignalP.^3^ The main structure of the amino acid sequence and the predicted protein molecular weight were obtained with the EXPASY ProtParam tool.^4^ The genes of this enzyme were further contrasted with the National Center for Biotechnology Information non-redundant (NR) protein database utilizing the NCBI BLASTp algorithm, and a phylogenetic tree built on the maximum likelihood method was constructed employing MEGA 7.0. Autogenetic test bootstrap values are repeatedly labeled 1,000 times on the branches to evaluate the system’s phylogenetic tree confidence.

### Heterologous expression and purification of recombinant β-glucosidase

A sole colony of the recombinant strain *E. coli* BL21(DE3)-*lqbg8* was selected and inoculated in LB liquid medium containing 50 μg/mL of kanamycin. The strain was cultured overnight at 37°C and 220 rpm for overnight incubation. Then the above culture was incubated in 200 mL LB medium containing 50 μg/mL kanamycin at 1% inoculum, 37°C, 220 rpm for about 2.5 h. Until OD600 ≈ 0.6, IPTG was added to a final concentration of 0.5 mM to induce expression of the recombinant β-glucosidase. Cultures were incubated at 25°C and 220 rpm for 6 h. Finally, cultures were centrifuged at 4°C and 7,000 rpm for 20 min to obtain *E. coli* cells.

The collected *E. coli* cells were resuspended in centrifuge tubes with 30 mL PBS solution (pH 7.6) containing 10 mM imidazole, placed in an ice-water mixture and crushed with an ultrasonic cell crusher for 30 min (Power 200w, 5 times/min), followed by centrifugation as shown above. The supernatant was collected for protein purification. The Ni-NTA column was equilibrated with 10 mL equilibration solution (PBS solution with 10 mM imidazole, pH 7.6). The supernatant was added to the Ni-NTA column for loading and repeated once, after which the column was washed with 100 mL equilibration solution. The target protein was eluted with 10 mL eluent solution (PBS solution containing 250 mM imidazole, pH 7.6), and the purified protein was collected in 1.5 mL centrifuge tubes with 1.0 mL per tube. The protein concentration was estimated by Bradford assay, using bovine serum albumin as standard (Bradford, 1976). The absorption value was measured at OD595 and protein size and complexity was analyzed using SDS-PAGE electrophoresis.

### Enzymatic properties of recombinant β-glucosidase LQBG8

#### Enzyme activity assay

The β-glucosidase activity assay was done with cellobiose as a substrate. The reaction mixture (100 μL) consisted of 90 μL of 1% (w/v) cellobiose dissolved in sodium phosphate disodium citrate buffer (pH 7.0, 0.2 mol/L) and 10 μL of enzyme (0.79 μg). and incubate at optimal temperature for 30 min, added 10 μL of reaction mixture to the 96-well plate and added 200 μL of glucose oxidase-peroxidase detection kit buffer (Biosino Bio-Technology & Science ING, China). After incubation at 37°C for 10 min, the absorption value was determined at 492 nm with a microplate reader. One unit (U) is defined as the amount of enzyme required to release 2 μmol of glucose from cellobiose per minute. Three replicates were done for each assay.

#### Determination of optimum temperature and thermal stability

The determination of the optimal temperature for β-glucosidase LQBG8 involved assessing its relatively activity across a range of temperatures (25 ~ 95°C) under sodium hydrogen phosphate-citric acid buffer (pH 7.0, 0.2 mol/L) refer to the “Enzyme activity assay” section for methodologies. The aim was to pinpoint the temperature at which the enzyme exhibits maximal activity. Subsequently, to evaluate its thermal stability, the purified β-glucosidase LQBG8 was subjected to incubation in three temperatures environments (65, 70, and 75°C) over durations of 0, 20, 40, 60, 80, 100, and 120 min. A control incubation period of 0 min was also employed (set at 100% activity). The residual enzyme activity was then quantified under standardized conditions.

#### Determination of optimum pH and pH tolerance

The determination of the optimal pH for β-glucosidase LQBG8 entailed assessing its enzymatic activity within buffers characterized by varying pH levels (ranging from pH 3.0–10.0, 0.2 mol/L) while maintaining optimal temperature conditions. The objective was to identify the pH value at which the enzyme exhibits peak activity. For pH values within the range of pH 3.0–8.0, disodium hydrogen phosphate-citric acid buffers were employed, while pH 8.0–10.0 was managed using glycine-sodium hydroxide buffers. To evaluate the enzyme’s pH tolerance stability, a ratio of 1:2 was utilized to combine and incubate 50 μL the purified β-glucosidase LQBG8 with buffer solutions that 100 μL different pH values. This mixture was subsequently subjected to a 4°C incubation for durations of 12 and 24 h. Following incubation, the residual enzyme activity was determined under standardized conditions.

### Effects of metal ions, chemical reagents and ionic liquids on enzyme activity

To assess the impact of metal ions, chemical reagents, and ionic liquids on β-glucosidase activity, a series of experiments were conducted. Different metal ions (including K^+^, Mg^2+^, Fe^3+^, Ca^2+^, Zn^2+^, Co^2+^, Cu^2+^, Ag^+^, Mn^2+^, Pb^2+^, and Ni^2+^) were incrementally introduced into the reaction system to attain concentrations of both 1 mM and 10 mM. Various chemical reagents, such as disodium ethylenediaminetetraacetic acid (EDTA), sodium dodecyl sulfate (SDS), phenylmethylsulfonyl fluoride (PSMF), and dithiothreitol (DTT), were similarly added at concentrations of 0.1 and 1%. Furthermore, the ionic liquid chosen for investigation, 1-ethyl-3-methylimidazole acetate, was incorporated into a sodium hydrogen phosphate-citric acid buffer (pH 5.6, 0.2 mol/L) solution. This mixture was then combined with the substrate, resulting in a final concentration of 1 and 10%. Subsequently, enzyme activity was evaluated under standardized conditions to discern the effects of these additions on the enzymatic activity of β-glucosidase.

### Effect of glucose on enzyme activity

Based on the standard enzymatic reaction system, using *p*-nitrophenyl-β-D-glucopyranoside (pNPG) as the substrate, 10 μg of protein was added to a 150 μL reaction mixture containing 1.0 mM pNPG (Sigma, St. Louis, MO, United States). Following an incubation period of 5 min at the optimal temperature, the reaction was terminated by adding 450 μL of 1 M Na_2_CO_3_. The subsequent quantification of *p*-nitrophenol (pNP) release was achieved by comparing to the absorbance of a pNP (Sigma, United States) standard curve. The measurement entailed monitoring the absorbance at 405 nm. In investigating the influence of D-glucose on LQBG8’s catalytic activity, distinct concentrations of D-glucose (ranging from 0 to 3 M) were added to the reaction system. This facilitated the measurement of relative enzymatic activity. Notably, a control group lacking glucose was incorporated for comparative analysis. *K_i_* value is calculated by formula, *K_i_* = IC_50_/(1 + [S]/*K_m_*).

### Determination of substrate specificity

In order to elucidate the substrate specificity of LQBG8, a range of substrates were employed, including cellobiose, lactose, *p*-Nitrophenyl-β-D-glucopyranoside (pNPG, 1 mM), *p*-Nitrophenyl-β-D-galactopyranoside (1 mM), β-gentiobiose, d-(+)-maltose monohydrate, d-(+)-trehalose dihydrate, d-(+)-melibiose hydrate, sucrose, wheat bran xylan, corn cob xylan, bagasse xylan, sodium carboxymethylcellulose (CMC), and Avicel, all substrates were purchased from China Biotechnology Company. Unless otherwise specified, one of them was utilized as a substrate at a concentration of 1% (w/v) to quantitate enzyme activity. The assessment of cellulase and xylanase activity was executed through a 3,5-dinitrosalicylic acid assay.

### Determination of kinetic parameters

The kinetic parameters (*K*_m_ and *V*_max_) of LQBG8 were ascertained through reactions involving various cellobiose concentrations (ranging from 0 to 20 mg/mL). Prior to determination, both 90 μL substrate and 10 μL enzyme (0.79 μg) were subjected to preheating at 70°C for 1 min. These reactions were conducted over a 10-min duration under standard conditions (70°C, pH 5.6, and 0.79 μg purified recombinant protein). The calculation of *K*_m_ and *V*_max_ values was accomplished using the Lineweaver-Burk graph methodology.

### Determination of hydrolysis of corn stover by LQBG8

Ten grams of corn stover (ZHENDAN 958 maize, variety approval number 20000009) were subjected to crushing with a grinder, followed by sieving through an 80-mesh sieve. The resulting material was then boiled in 100 mL of hot water for a duration of 30 min. Subsequently, filtration was performed using filter paper, and the obtained filtrate was dried at 80°C. A total of 0.2 g of hot water-pretreated corn stover was introduced into 1 mL of buffer solution (pH 5.6). To this, 0.2 mg of cellulase (Sangon Biotech, China) obtained from *Trichoderma reesei* and 0.02 mg of LQBG8 were added to form the reaction system. Mixtures were incubated at 50°C. Samples were extracted from the reaction system at specific time intervals: 0, 1, 2, 3, 4, 5, 6, 7, 8, 18 and 24 h from the commencement of the reaction. The glucose concentration within the reaction solution was determined through employment of a glucose oxidase detection kit. The control group consisted of the reaction solution without the addition of enzymes. Repetition of each experimental group was carried out three times, and the mean value was derived for analysis.

### Homology modeling and kinetic simulation of β-glucosidase

A closely related enzyme to LQBG8 was identified through the construction of a phylogenetic tree, finding related proteins that share a common lineage to a normal-temperature β-glucosidase (ThBGL1A) sourced from *Thermobifida halotolerans.* Utilizing the SWISS-MODEL server,^5^ a homology model for LQBG8 was developed. The template structure was derived from the GH1 family β-glucosidase of *Tepidiforma bonchosmolovskayae*, which exhibited the highest similarity to LQBG8. Similarly, ThBGL1A underwent homology modeling using the β-glucosidase from *Thermobifida cellulosilytica* TB100, possessing the highest similarity to ThBGL1A, as the template structure.

The procedures and parameters for kinetic simulation of both LQBG8 and ThBGL1A were maintained identically. Armed with the generated protein structure and topology files, molecular dynamics (MD) simulations were conducted using the GROMACS software (Abraham et al., 2015). The GROMACS software, specifically the “gmx rms” tool, was employed to calculate and compare the root mean square deviation (RMSD) of the dynamic structure concerning the initial structure throughout the MD simulation. Additionally, the root mean square fluctuation (RMSF) of each residue’s Cα was determined using the same software tool.

### Statistical analysis

Unless otherwise stated, all assays were carried out in triplicate and the average used in all analyses. The results were analyzed by SPSS 20.0 and expressed as means ± SEM (standard error of mean). Statistical analyses were performed by using the T-test to compare the treated and untreated groups. In all comparisons, *p* values <0.05 were considered statistically significant.

## Results and discussion

### Cloning, expression, purification and sequence analysis of LQBG8

**Figure 1:**
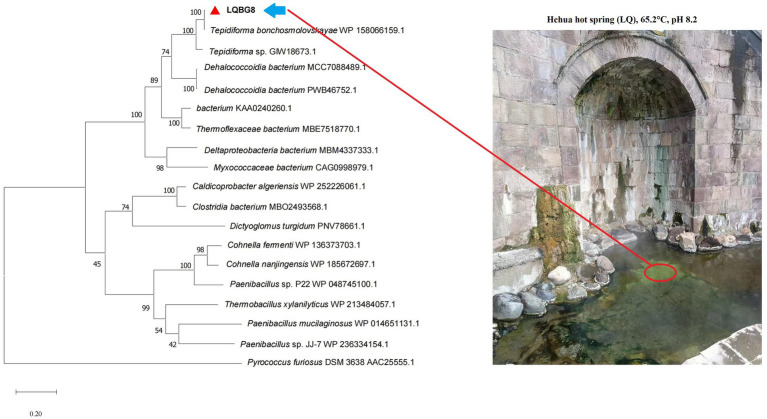
Phylogenetic dendrogram obtained by maximum likelihood analysis based on amino acid sequences showing the phylogenetic position of LQBG8 related to β-glucosidases. Bootstrap values (expressed as a percentage of 1,000 replications) are given at nodes.

LQBG8 from the hot spring soil metagenome encodes a sequence of 446 amino acids, and its corresponding nucleotide sequence has been uploaded to the NCBI database with accession number OP880885. No signal peptide sequence was found. The anticipated theoretical values for the isoelectric point (*pI*) and molecular weight (*Mw*) are 5.6 and 50.1 kDa, respectively. Analysis of LQBG8’s amino acid sequence revealed its closest resemblance (99.78%) to the β-glucosidase (NCBI: WP158066159.1) within the GH1 family from *Tepidiforma bonchosmolovskayae*. The difference between the two protein sequences is that the 48th amino acid residue of LQBG8 is Ser, while the other is Gly. Phylogenetic analysis further corroborated the similarity between LQBG8 and the aforementioned β-glucosidase from *Tepidiforma*, with the two enzymes clustering together within a branch (refer to Figure 1). Notably, *Tepidiforma bonchosmolovskayae* is a recently discovered thermophilic strain sourced from hot springs (Kochetkova et al., 2020), therefore, it is likely that LQBG8 is also a thermophilic enzyme.

**Figure 2:**
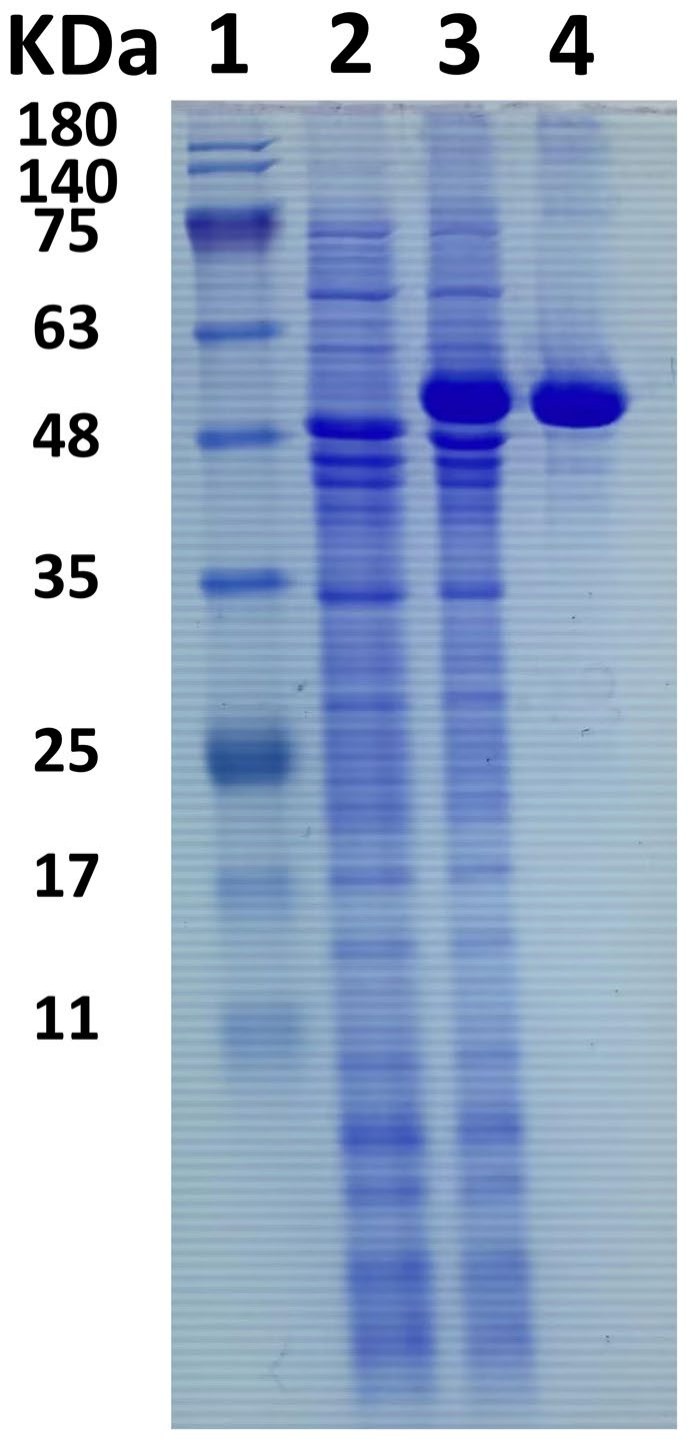
SDS-PAGE electrophoresis of β-glucosidase LQBG8. Lane 1, protein molecular weight marker, mass indicated on the left; lane 2, total protein in no-induced *Escherichia coli* BL21/*pET28a-lqbg8*; lane 3, total protein in IPTG-induced *E. coli* BL21/*pET28a-lqbg8*; lane 4, purified LQBG8.

An in-depth sequence analysis confirms that the anticipated enzyme protein of *LQBG8* comprises the catalytic module of glycoside hydrolase family 1 (GH1). Notably, successful expression of *LQBG8* was achieved in *E. coli* BL21(DE3), followed by purification through Ni-NTA affinity chromatography, leading to the isolation of the recombinant protein’s N-terminal, which was tagged with His. The application of SDS-PAGE analysis revealed a congruence between the molecular weight of the recombinant β-glucosidase protein and its theoretical projection, with the observed band size of approximately 50 kDa (see Figure 2).

### Enzymatic properties of recombinant β-glucosidase LQBG8

#### Optimum temperature and thermal stability

**Figure 3:**
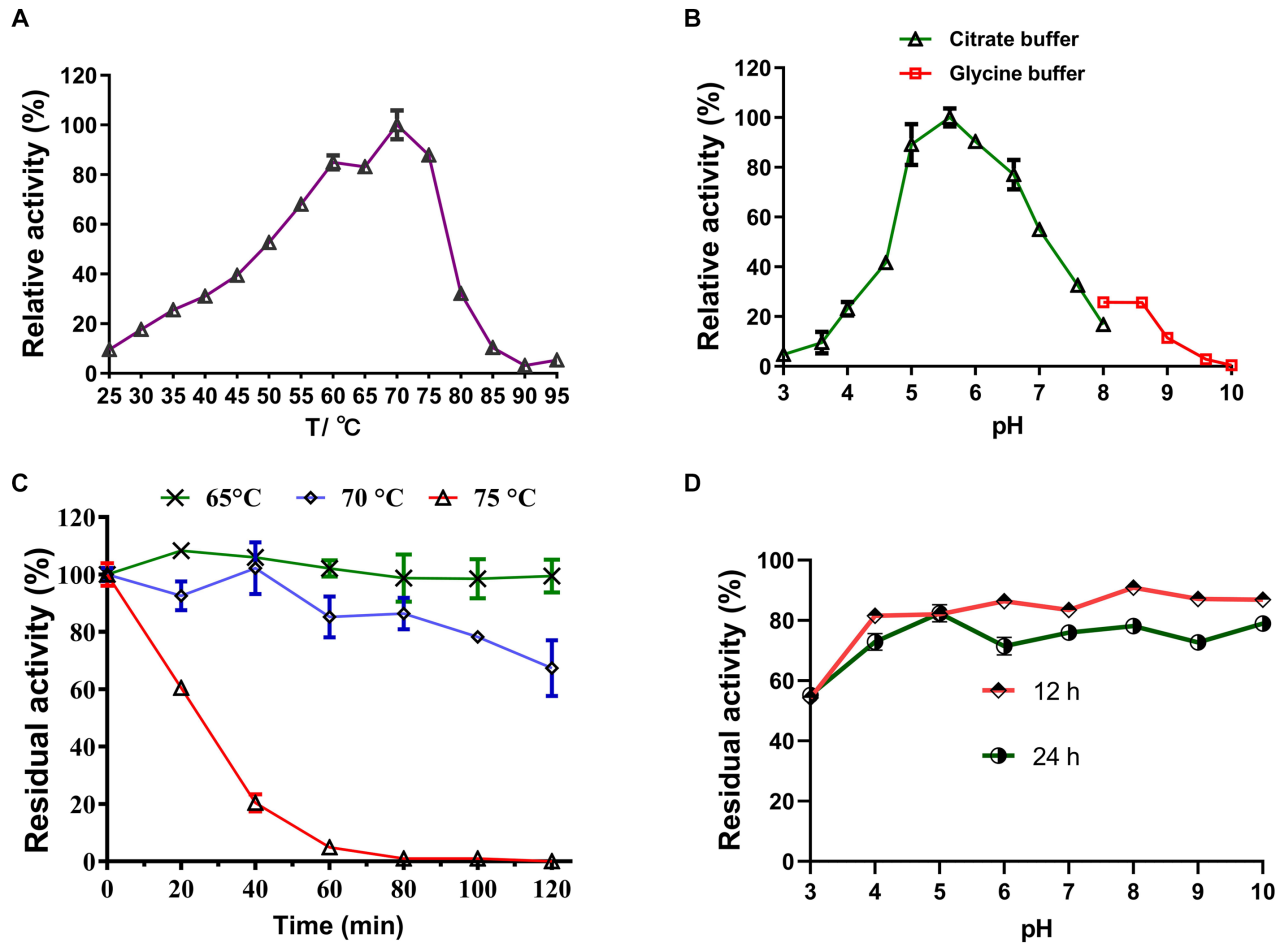
Effects of temperature and pH on the activity and stability of the recombinant LQBG8. **(A)** Temperature effect on the activity of LQBG8. **(B)** pH effect on the activity of LQBG8. **(C)** The effect of temperature on stability at different temperatures (65, 70 and 75°C) for 0, 20, 40, 60, 80, 100, and 120 min. **(D)** The effect of pH on stability. Values represent the mean of three biological replicates. Error bars represent the mean ± SEM of three biological replicates. The primary activity was taken as 100%.

Under optimal pH conditions, LQBG8 exhibited its highest activity at 70°C (see Figure 3A). Noteworthy parallels exist among the reported β-glucosidases with a 70°C optimum temperature, such as ASG isolated from apple seeds (Yu et al., 2007), the heat-resistant β-glucosidase BGL I derived from *Periconia* sp. (Harnpicharnchai et al., 2009), and the β-glucosidase (BGLA) originating from *Aspergillus* documented by Li et al. (2018), among others. An array of β-glucosidases optimized for distinct temperatures have also been documented. For instance, the temperature optimum for a thermophilic β-glucosidase produced by the rhizobacterial strain LSKB15, isolated from desert environments, stood at 60°C (Ahmed et al., 2018). Similarly, β-glucosidases from *Flammulina velina* and *Flammulina velutipes* exhibited an optimum temperature of 50°C (Daroit et al., 2008; Mallerman et al., 2015). Within a span of 2 h, LQBG8 demonstrated steadfast activity, retaining approximately 100% of its functionality at 65°C. As the temperature elevated to 70°C, the activity gradually diminished, yet remained above 60%. Upon reaching 75°C, the activity exhibited a rapid decline, and its half-life (t_1/2_) was measured at 45 min (Figure 3C). These outcomes collectively underscore the robust thermal stability exhibited by LQBG8. This distinctive feature renders LQBG8 applicable across diverse domains, including papermaking, laundry, and animal feed (Gomes et al., 2000).

#### Optimum pH and pH-tolerant stability

Under optimal temperature conditions, the optimal reaction pH for LQBG8 was determined to be 5.6, and exhibited over 95% relative activity at pH 5.6–6.6 (as depicted in Figure 3B). This closely aligns with the findings associated with β-glucosidase (CelB) derived from *Pyrococcus furiosus*, which also manifests an optimal pH value of 6.0. Furthermore, CelB displays reduced activity, accounting for <30%, at pH extremes such as 4.0 or 9.0 (Li et al., 2013). Notably, LQBG8 exhibited sustained stability across a pH spectrum of 4.0 to 10.0. Remarkably, after incubation within a buffer at 4°C for both 12 and 24 h, LQBG8 retained over 70% of its residual enzyme activity (Figure 3D). These outcomes underscore the substantial pH tolerance range and robust stability characteristic of LQBG8. It has been previously documented that β-glucosidases capable of maintaining their activity across a broad pH range play a pivotal role in enhancing the conversion efficiency of cellulosic materials into glucose. Consequently, pH stability stands as another vital attribute influencing the advancement of β-glucosidase productivity (Jiang et al., 2011; Xia et al., 2016).

### Effects of metal ions, chemical reagents and ionic liquids on enzyme activity

**Figure 4:**
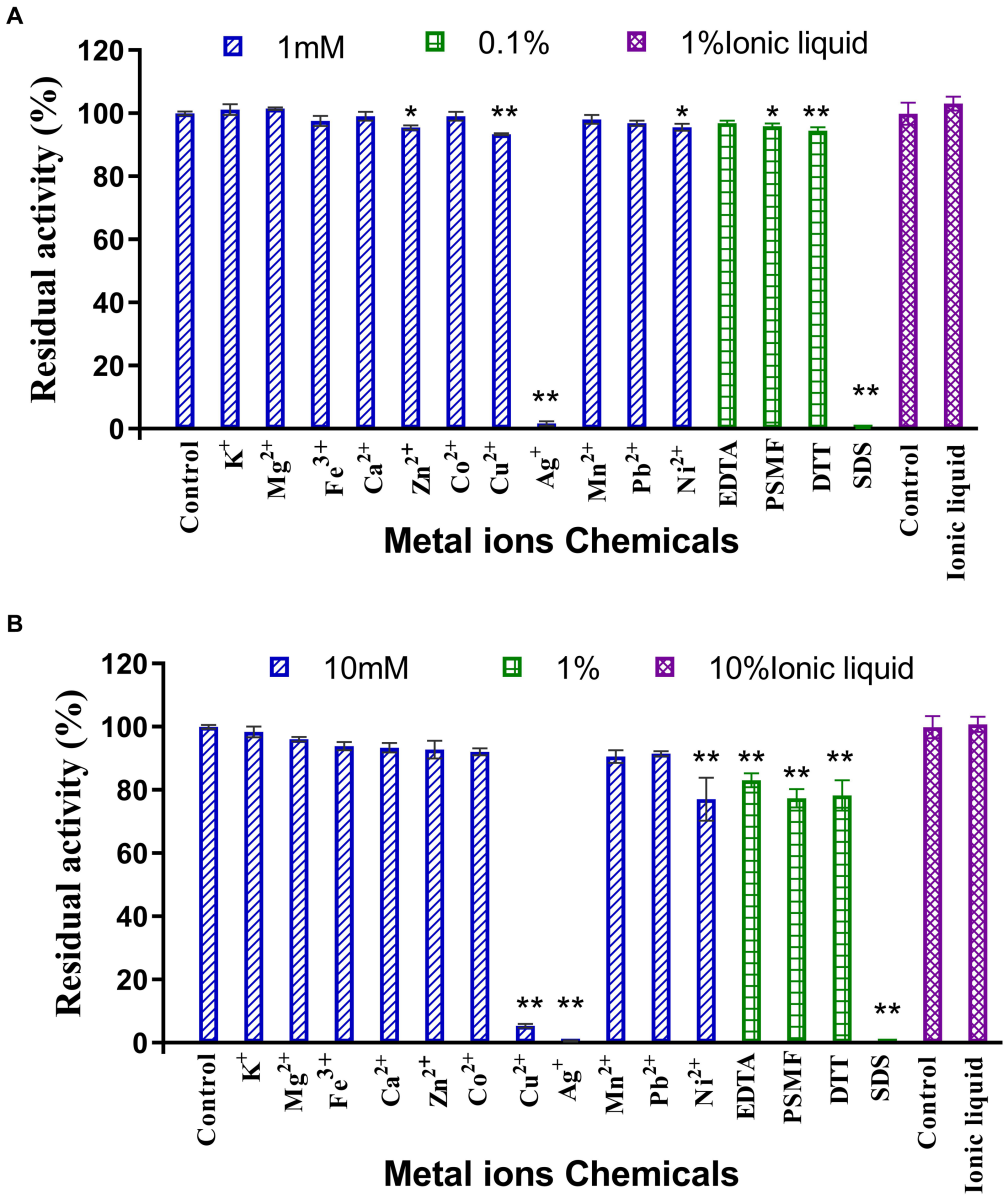
Effect of metal ions and chemicals on recombinant LQBG8 activity. **(A)** The effect of 1 mM metal ions, 0.1% inhibitors and 1% ionic liquid on the activity of LQBG8. **(B)** The effect of 10 mM metal ions, 1% inhibitors and 10% ionic liquid on the activity of LQBG8. Values represent the mean of three biological replicates. Error bars represent the mean ± SEM of three biological replicates. ^**^p < 0.01, ^*^p < 0.05.

The results of the assessment on the impact of metal ions and chemical reagents upon LQBG8 enzyme activity are presented in Figure 4. Notably, the presence of 1 mM and 10 mM concentrations of K^+^, Mg^2+^, Fe^3+^, Ca^2+^, Co^2+^, Mn^2+^, and Pb^2+^ metal ions exhibited negligible influence on the activity of LQBG8. This observation underscores the inherent stability of LQBG8’s activity, as the majority of ions had minimal impact, with activity remaining robust (>85%). Significantly, chemical reagents in the form of SDS at concentrations of 0.1 and 1% exerted pronounced inhibitory effects on LQBG8 activity. Conversely, other reagents at a concentration of 0.1% demonstrated insignificant effects on LQBG8 enzyme activity. At 1%, a slight inhibitory effect was observed. Notably, the metal chelator EDTA displayed no inhibitory effect, affirming that LQBG8 operates independently of metal ions. The presence of DTT, a common protein stabilizing agent for those containing free sulfhydryl groups, failed to elicit change, indicating the absence of disulfide bonds in LQBG8’s structure and thus, maintaining its enzyme activity (Yin et al., 2020). The anionic surfactant SDS, having a strong influence, essentially reduced LQBG8 activity to zero. This outcome is attributed to SDS interfering with hydrophobic interactions and inducing a structural unfolding of the protein. Conversely, the presence of 1 and 10% ionic liquid did not significantly affect enzyme activity, indicating LQBG8’s tolerance to ionic liquid. Notably, at a 1 mM concentration, Cu^2+^ had negligible impact on LQBG8 enzyme activity, whereas at 10 mM, its inhibitory effect mirrored that of Ag^+^ and SDS. This aligns with observations in β-glucosidase from *Thermomucor indicaeseudaticae* and marine microbial metagenomic Bgl1A (Fang et al., 2010).

### Effect of glucose concentration on enzyme activity

**Figure 5:**
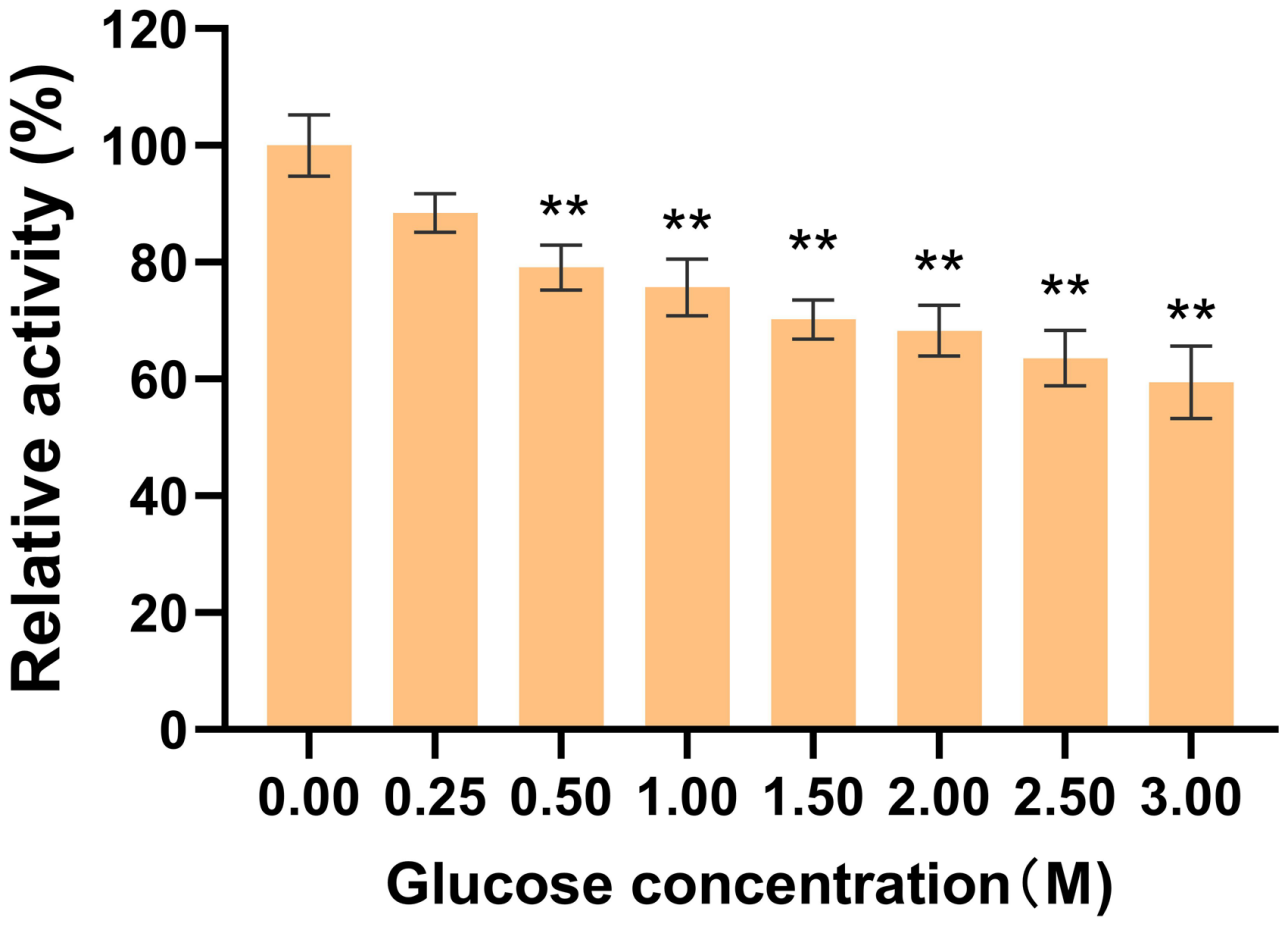
Effect of glucose on the activity of recombinant LQBG8. Values represent the mean of three biological replicates. Error bars represent the mean ± SEM of three biological replicates. ^**^p < 0.01, ^*^p < 0.05.

The assessment of glucose tolerance in LQBG8 employed 1.0 mM pNPG as the substrate. Setting glucose-free conditions as the control group (Figure 5), it is discerned that upon reaching a glucose concentration of 1 M, the relative enzyme activity surpassed 70%. Similarly, at a glucose concentration of 3 M, the relative enzyme activity maintained a level of 60%. The kinetic parameters of LQBG8 were characterized using pNPG as substrate, with *K_m_* and *V_max_* values of 0.26 ± 0.01 mM and 143 ± 1.2 μmol/min/mg, respectively. The *K*_i_ value of LQBG8 for glucose was determined to be about 824.7 mM. Existing research has established that the majority of β-glucosidases exhibit heightened sensitivity to glucose, with their *K*_i_ values ranging between 0.5 mM and 100 mM. A value exceeding 200 mM typically signifies a noteworthy tolerance to glucose (Yin et al., 2021). LQBG8 surpasses many of the previously reported glucose-tolerant β-glucosidases in its resistance to glucose. By comparison, when the glucose concentration for BcBgl1A from *Bacillus cellulosilyticus* reaches 1 M, its relative enzymatic activity plummets to <10% (Wu et al., 2018). Similarly, the *K*_i_ value of 150 mM for the β-glucosidase TnBglB, cloned from the hyperthermophilic *Thermotoga naphthophila* RKU-10 T, exemplifies the sensitivity of some enzymes to glucose (Akram et al., 2018). In another example, the GH1 β-glucosidase from *Exiguobacterium antarcticum* B7 experiences a rise in relative enzyme activity to 135% upon glucose concentration reaching 200 mM, gradually decreasing afterward. Remarkably, when the glucose concentration attains 1 M, the relative enzyme activity remains close to 50%. Collectively, these findings underscore the robust glucose tolerance of LQBG8, which in turn holds promising applications within the high-sugar industry.

### Substrate specificity and kinetic constants of LQBG8

**Figure 6:**
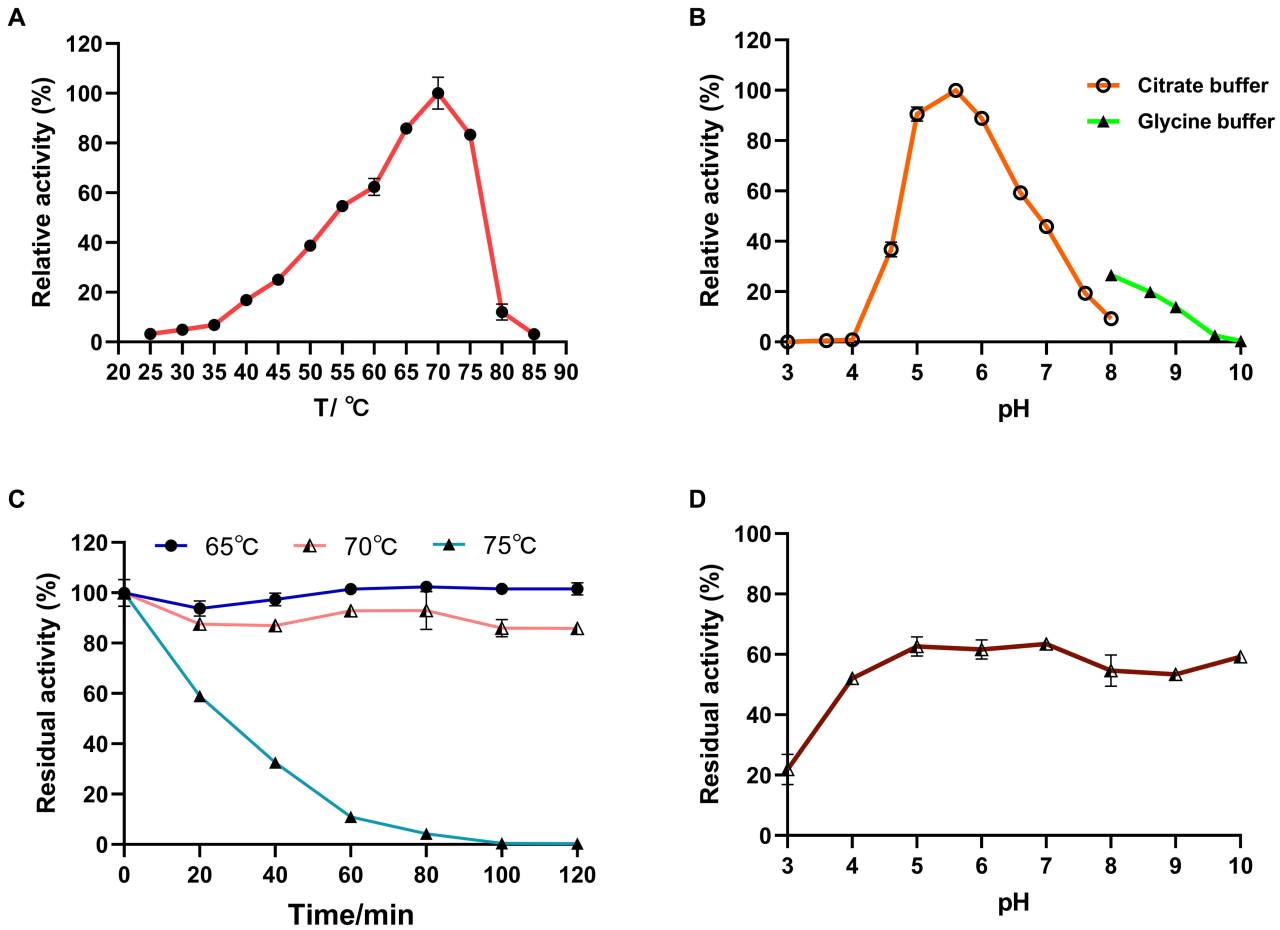
Effects of temperature and pH on the activity and stability of recombinant LQBG8 using lactose as substrate. **(A)** Temperature effect on the activity of LQBG8. **(B)** pH effect on the activity of LQBG8. **(C)** The effect of temperature on stability at different temperatures (65, 70 and 75°C) for 0, 20, 40, 60, 80, 100, and 120 min. **(D)** The effect of pH on stability. Values represent the mean of three biological replicates. Error bars represent the mean ± SEM of three biological replicates. The primary activity was taken as 100%.

Through the evaluation of LQBG8’s substrate specificity, the findings have revealed a broad spectrum of substrate hydrolysis capabilities (as outlined in Table 1). Notably, LQBG8 demonstrated hydrolytic activity towards various substrates, including cellobiose (11.51 ± 0.08 μmol/min/mg), lactose (8.40 ± 0.06 μmol/min/mg), *p*-Nitrophenyl-β-D-glucopyranoside (3.22 ± 0.14 μmol/min/mg), *p-*Nitrophenyl-β-D-galactopyranoside (2.50 ± 0.04 μmol/min/mg) and β-gentiobiose (5.04 ± 0.41 μmol/min/mg). Conversely, it exhibited no hydrolytic activity towards d-(+)-maltose monohydrate, d-(+)-trehalose dihydrate, d-(+)-melibiose hydrate, sucrose, xylan, CMC, and Avicel. In order to further determine the hydrolysis of LQBG8 on lactose, the effects of temperature and pH on the activity and stability of recombinant LQBG8 were determined using lactose as the substrate. The results are shown in Figure 6. The results are consistent with those measured using cellobiose as the substrate. These results underline LQBG8’s classification as a multifunctional glycoside hydrolase, concurrently possessing β-glucosidase and β-galactosidase activities. Remarkably, this marks instance wherein a GH1-derived β-glucosidase is also discovered to possess β-galactosidase activity. Analogously, Deng et al. (2020) reported the GH1-derived β-glucosidase (BglD1) from the deep-sea bacterium *Bacillus* sp. D1 to exhibit β-galactosidase activity, thus rendering it a multifunctional enzyme. Akin findings are echoed by Ariaeenejad et al. (2023), who unveiled a novel recombinant bifunctional enzyme (xylanase/β-glucosidase), PersiBGLXyn1, within the human bovine rumen metagenome. These observations accentuate the enhanced utility of such bifunctional enzymes (Bao et al., 2012; Ariaeenejad et al., 2021).

**Table 1:** Substrate specificities of LQ-BG8.

Substance	Special activity (μmol/min/mg)
Cellobiose	11.51 ± 0.08
Lactose	8.40 ± 0.06
*p*-Nitrophenyl-β-D-glucopyranoside	3.22 ± 0.14
4-Nitrophenyl-β-D-galactopyranoside	2.50 ± 0.04
β-gentiobiose	5.04 ± 0.41
D-(+)-maltose monohydrate	0
D-(+)-trehalose dihydrate	0
D-(+)-melibiose hydrate	0
Sucrose	0
Wheat bran xylan	0
Corn cob xylan	0
Bagasse xylan	0
CMC	0
Avicel	0

Based on the Michaelis–Menten equation for cellobiose, the kinetic properties of LQBG8 were deduced, and the Lineweaver-Burk and Hanes-Woolf plots were constructed (refer to Supplementary Figures S1, S2). Under optimal temperature and pH conditions, the calculated values for *K*_m_ and *V*_max_ were determined to be 28 ± 1.9 mg/mL and 55 ± 3.2 μmol/min/mg, respectively. Notably, LQBG8’s *K*_m_ value surpasses that of previously reported Bgl1973 from *Leifsonia* sp. ZF2019 (He et al., 2022), β-glucosidases originating from *Thermococcus* sp. (Sinha and Datta, 2016), and β-glucosidases derived from *Scytalidium thermophilum* (Zanoelo et al., 2004). This outcome suggests LQBG8’s heightened affinity for cellobiose, a prominent substrate among most microbial β-glucosidases.

### Determination of hydrolysis of corn stover by LQBG8

**Figure 7:**
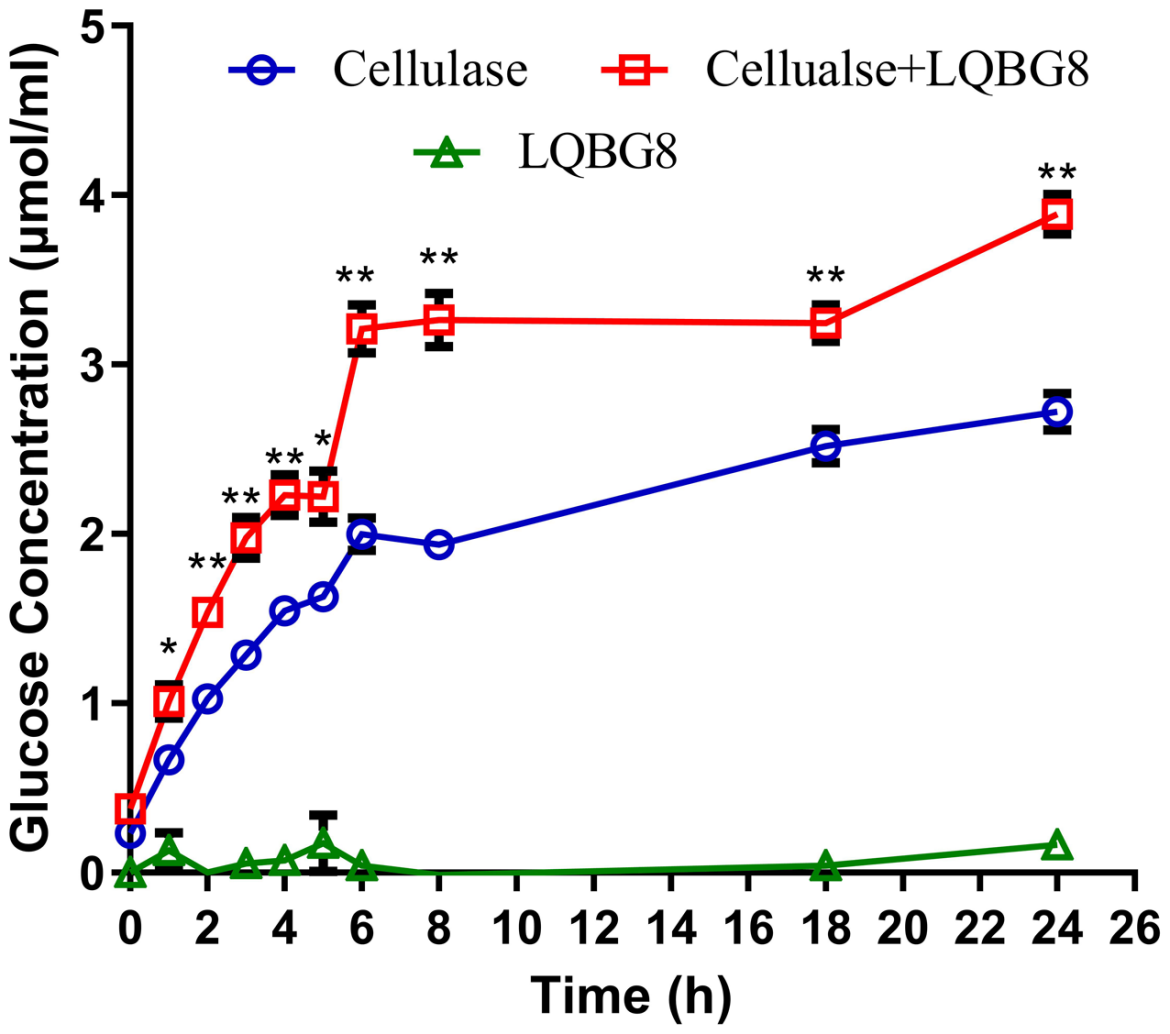
Cooperation of LQBG8 with the commercial cellulase in corn stover degradation. The green triangle showed the result of LQBG8 hydrolyzing corn stover. The blue circle showed the results of commercial cellulase hydrolysis of corn stover. The red rectangle represented the result of LQBG8 and commercial cellulase synergistic hydrolysis of corn stover. Values represent the mean of three biological replicates. Error bars represent the mean ± SEM of three biological replicates. ^**^p < 0.01, ^*^p < 0.05.

Assessing the enzymatic hydrolysis of corn stover by LQBG8 serves to effectively appraise its potential utility in lignocellulose degradation. The results are delineated as follows (refer to Figure 7). The introduction of LQBG8 into the commercial cellulase system yielded a discernible enhancement in reaction rate and glucose yield. Following a 24-h duration, in comparison to the degradation rate exhibited by commercial cellulase in isolation (set as 100%), the degradation rate facilitated by LQBG8 exhibited a remarkable escalation of 43.7%. This marked augmentation underscores the considerable efficacy of LQBG8 in tandem with commercial cellulase, thereby affirming its tangible contributions to the enhancement of lignocellulose degradation. It is noteworthy that LQBG8 did not exhibit autonomous hydrolytic activity on corn stover. Nevertheless, it manifested a notable synergistic effect when co-applied with commercial cellulase. This synergistic outcome further attests to LQBG8’s intrinsic worth in augmenting the process of lignocellulose degradation.

### Homology modeling of LQBG8 and ThBGL1A

**Figure 8:**
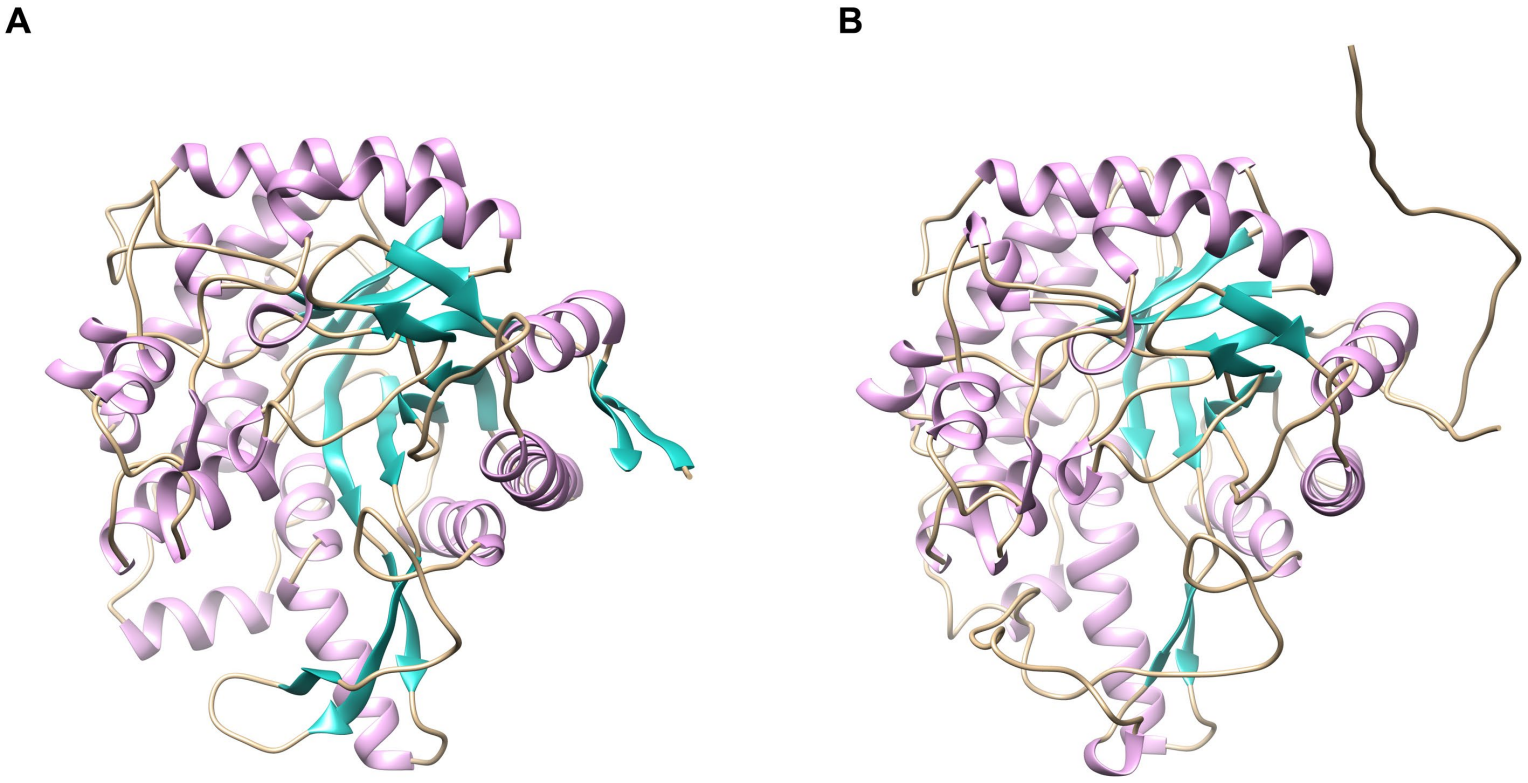
Homology modeling of LQBG8 **(A)** and ThBGL1A **(B)** protein structures.

Homology modeling-driven structural analysis reveals that the thermophilic enzyme LQBG8 investigated in this study, alongside the normal temperature β-glucosidase from *Thermobifida halotolerans*, both assume monomeric configurations (depicted in Figure 8). Notably, the static structures of these two enzymes exhibit a striking resemblance. This aligns with existing literature, wherein other thermostable β-glucosidases derived from GH1 also exhibit monomeric structures within aqueous environments (Colussi et al., 2015), suggesting that homology modeling of LQBG8 structure holds certain reference value for investigating its thermophilic mechanism.

### Structural fluctuations during MD simulations

**Figure 9:**
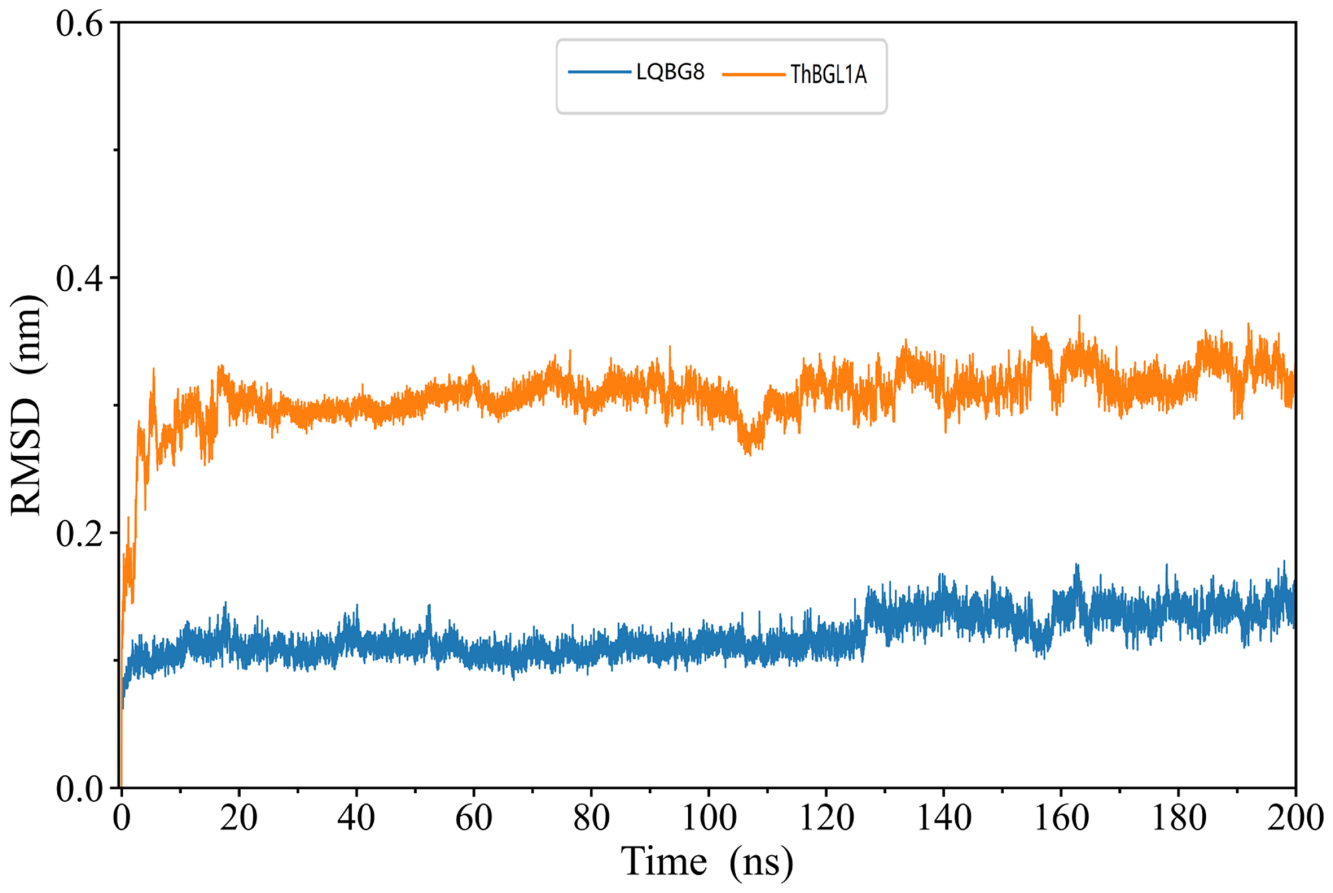
LQBG8 (blue line) and ThBGL1A (orange line) are the RMSD value variation curves of the skeleton atoms as a function of time in MD simulations.

The skeleton RMSD, as a function of time, was first calculated for both simulated systems, serving to assess the overall equilibrium and stability of LQBG8 and ThBGL1A throughout the simulation. As depicted in Figure 9, the skeleton RMSD for the LQBG8 simulation system attained a relatively stable value within approximately 10 ns, subsequently maintaining a consistent level of fluctuation. Conversely, the skeleton RMSD for the ThBGL1A simulation system took roughly 20 ns to stabilize at a consistent value, followed by a similar pattern of stable fluctuations. Evidently, the overall RMSD value of ThBGL1A exceeds that of LQBG8 during the simulation, implying that ThBGL1A experiences more pronounced fluctuations and conformational alterations in comparison to its initial structure than observed with LQBG8. Importantly, LQBG8 consistently maintains a relatively stable conformation throughout the simulation, which may underpin its superior thermal stability.

### Analysis of conformational flexibility

**Figure 10:**
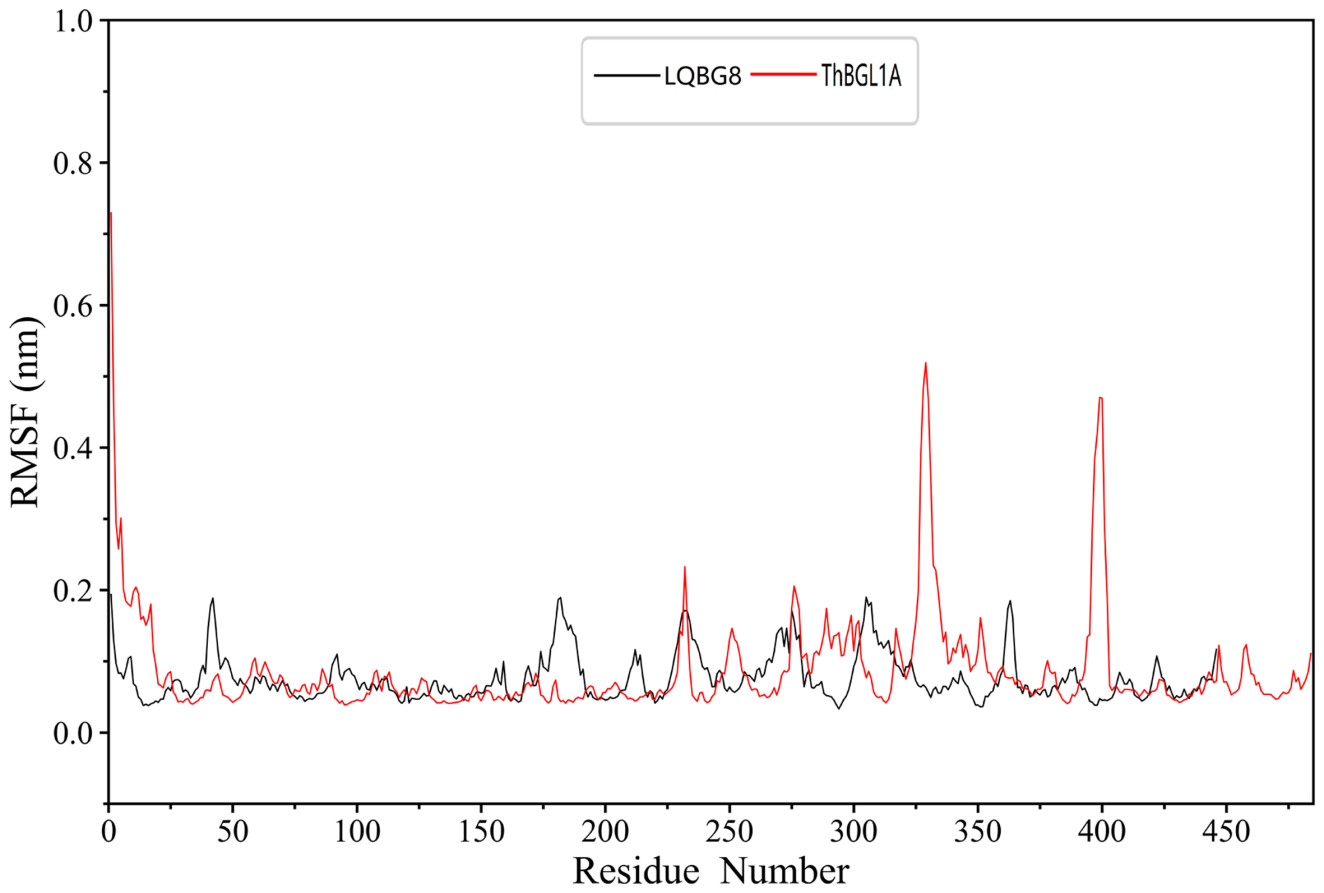
Root mean square fluctuation (RMSF) values of each residue Cα atom in LQBG8 (black line) and ThBGL1A (red line).

In order to explore the conformational dynamics exhibited by LQBG8 and ThBGL1A during the simulation, we conducted calculations to ascertain the RMSF of the Cα atoms throughout the simulation duration. As illustrated in Figure 10, upon a comparison of the derived RMSF values, it becomes evident that both LQBG8 and ThBGL1A manifest RMSF curves marked by analogous fluctuations, thereby suggesting a partial resemblance in the 3D structures and contact interfaces of these two proteins. Notably, the majority of regions within LQBG8 exhibit lower RMSF values in comparison to ThBGL1A. This observation aligns harmoniously with the contrast seen in the RMSD values between the two proteins. However, it is noteworthy that there exist two distinct loop regions spanning 325–350 and 390–410 amino acid residues, in which the RMSF values of ThBGL1A surpass those of LQBG8 to a considerable extent. This outcome serves to signify that ThBGL1A boasts a greater overall flexibility in comparison to LQBG8, further underscoring the discernible differences in their global dynamics.

## Conclusion

This study represents the inaugural instance of cloning a β-glucosidase gene, designated as LQBG8, belonging to the GH1 family, from the microbial metagenome sourced from the Tengchong Hehua hot spring. Subsequently, the gene was expressed heterologously in *E. coli* BL21(DE3), and the ensuing recombinant β-glucosidase LQBG8 was meticulously isolated and purified for comprehensive enzymatic property analysis. Notably, this investigation revealed the enzyme’s robust thermostability, glucose tolerance, and ionic liquid resistance. Furthermore, the enzyme demonstrated a synergistic effect with commercial cellulase, resulting in a 43.7% augmentation of sugar content following the hydrolysis of hot water-treated corn stover. Intriguingly, LQBG8 exhibited dual functionality, showcasing both β-glucosidase and β-galactosidase activities. Collectively, these findings underscore the exceptional specificity and attributes of LQBG8, positioning it as an optimal candidate enzyme for diverse biotechnological applications, particularly in the realms of cellulose degradation and bioethanol production.

## Data availability statement

The metagenomic data of Hehua hot spring presented in the study are deposited in the NCBI RefSeq repository, accession number PRJNA1015443.

## Author contributions

Y-YH: Investigation, Project administration, Writing – original draft. Z-HL: Data curation, Investigation, Methodology, Writing – original draft. H-ZZ: Resources, Writing – original draft. QZ: Investigation, Resources, Writing – original draft. M-TL: Data curation, Investigation, Methodology, Writing – original draft. PS: Data curation, Methodology, Writing – original draft. FW: Resources, Writing – original draft. DZ: Investigation, Writing – original draft. W-DX: Conceptualization, Validation, Writing – review & editing. Y-RY: Conceptualization, Funding acquisition, Supervision, Writing – original draft, Writing – review & editing.

## Footnotes

^1^https://img.jgi.doe.gov/cgi-bin/mer/main.cgi

^2^http://blast.ncbi.nlm.nih.gov/Blast.cgi

^3^http://www.cbs.dtu.dk/services/SignalP/

^4^http://web.expasy.org/protparam/

^5^https://swissmodel.expasy.org
