# Supplementary material for: Characterization of a thermophilic and glucose-tolerant GH1 β-glucosidase from hot springs and its prospective application in corn stover degradation
Source: Front Microbiol. 2023 Dec 21;14:1286682. doi: 10.3389/fmicb.2023.1286682 (PMC10764553; doi:10.3389/fmicb.2023.1286682)
Supplement: Supplementary file 1 [file Data_Sheet_1.PDF]

Supplementary materials

List of the supporting materials:

2 Figure: Supplementary Figure S1，

Supplementary Figure S2

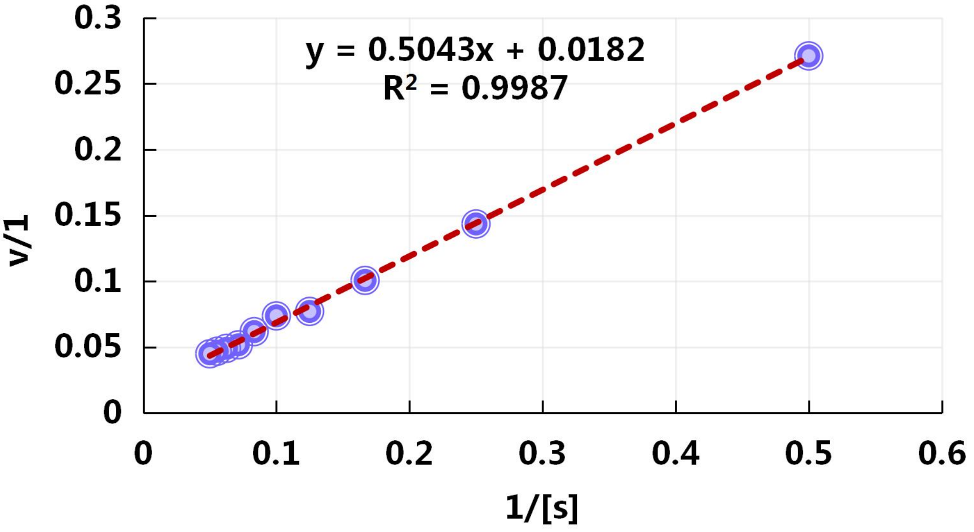

Supplementary Figure S1 Lineweaver-Burk plot of LQBG8 β-glucosidase

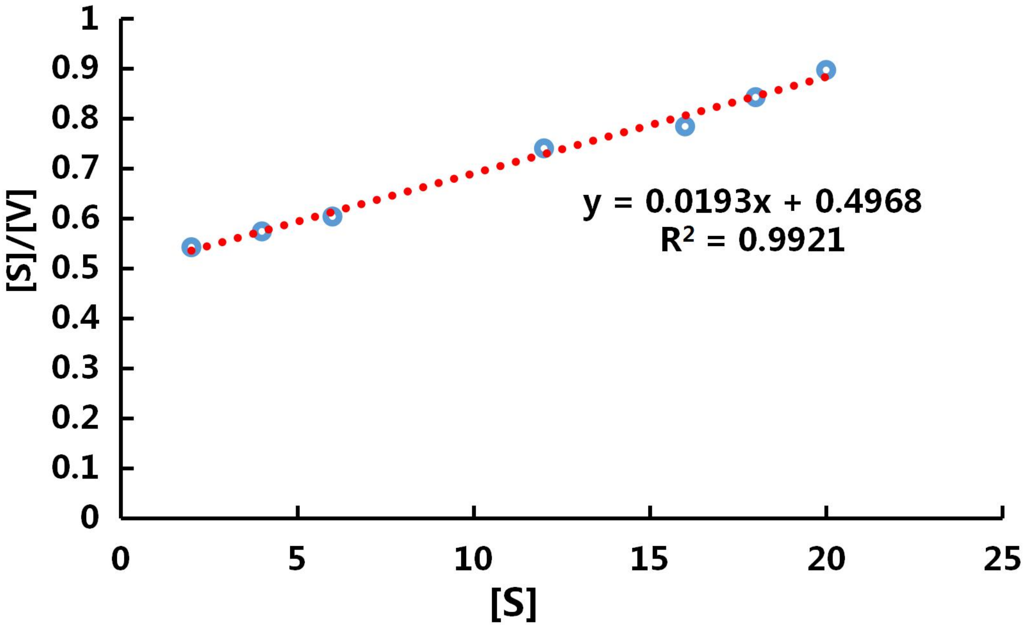

Supplementary Figure S2 Hanes-Woolf plot of LQBG8 β-glucosidase
